# On the Continued Fevers of Great Britain

**Published:** 1840-02-29

**Authors:** George C. Shattuck

**Affiliations:** Boston


### MEDICAL EXAMINER.

DEVOTED TO MEDICINE, SURGERY, AND THE COLLATERAL SCIENCES.

No. 9.] PHILADELPHIA, SATURDAY, FEBRUARY 29, 1840. [Vol. III.

### ON THE CONTINUED FEVERS OF GREAT BRITAIN.

By George C. Shattuck, Jr., M. D., of Boston.

Continued fever has been the subject of several works and memoirs within the last few years. Is it one disease? are its symptoms and lesions always the same? under what circumstances do they vary? Answers have been sought to these questions; and though much has been done, much remains to do. The disease prevails all over Europe, and over a great part of America. It is found amongst very different populations, in different climates. What is the influence of climate on its character and course? How far is it modified by natural and individual peculiarities? If information is to be had on these points, it will be found when a great number of exact and detailed observations shall have been carefully analyzed. Many must contribute their labours; and, considering the interest of the subject, and the present state of science, perhaps the little that I have to offer will not be without its value. I have thirteen observations. They were taken at the London Fever Hospital,—where, through the kindness of Dr. Tweedie, the attending physician, and of Mr. Goodfellow, the resident medical officer, every facility for the examination of the patients, and for anatomical researches, was afforded me.

The observations were taken at the request of M. Louis, who wished for further information on the question of the identity of continued fevers. Was it the same disease in England as in France, where, according to modern researches, the disease now exists only under one form, to which the name of typhoid fever has been given?

Dr. Gerhard has shown in his valuable papers published in the American Journal of Medical Sciences, that there are epidemics of fever at Philadelphia, where the disease is different in symptoms and lesions from the typhoid fever; and many of the French pathologists are disposed, with Dr. Gerhard, to recognise two diseases, the typhoid and the typhus. I have had some reference to this question in preparing this memoir.

Positive conclusions cannot be expected from so small a number of facts; but I have endeavoured to arrange and analyze them so that they may be easily understood and compared.

I shall give two of the observations; and, having called the attention of the reader to some of their prominent points, I shall proceed with a general account of symptoms and lesions. The facts having been submitted, every one can judge what conclusions should be drawn.

### FIRST OBSERVATION.

Mary Lembert, an Irish woman, unmarried, æt. twenty-two, a domestic, has lived at London for the last eleven years. Her hair chesnut, eyes blue, skin fair, stature and embonpoint not remarkable; her usual health good. Having been perfectly well during the preceding days, the 31st of January she was seized with chills. These were followed by heat, headach, thirst, loss of appetite, pains in the limbs and in the back. Her strength failed, her symptoms continued; and, since the 2d of February, she has kept her bed. There was no diarrhæa the first days of her illness; the 6th of February she took an emetic, the administration of which was followed by free and copious vomiting. She was brought to the fever hospital the 9th of February.

The 10th of February there was an expression of stupor in her countenance; the exercise of the mental faculties was somewhat difficult and irksome; her answers were slow, but correct and exact. She complained of headach, less severe than during the first days of her illness; of dizziness; the sight confused; the hearing was good; the eyes neither red nor suffused; the weakness such, that the patient stood up yesterday with great difficulty. The tongue was moist, covered with a whitish coat; she was thirsty, and had eaten nothing since the commencement of her illness. The abdomen was sonorous, somewhat distended, painful on pressure. She had taken no purgative, and had had three liquid stools since her entrance into the hospital. The pulse one hundred and four, sufficiently full; the heat increased. The respiration accelerated, easy. No eruption of any kind. ℞. Ol. Ricini ℥ij.

*Feb. 11th.*—The symptoms as yesterday. The cheeks red; the pulse one hundred; the prostration marked; the patient was quiet during the night.

The head was ordered to be shaved; compresses, dipped in cold water, to be applied to it; four leeches to be applied to the temples. Hyd. Cum. Creta gr. v, Pulv. Ipec. Comp. gr. iij, every four hours.

*Feb. 12th.*—The pulse one hundred, small, feeble, compressible; the skin hot, dry. The patient complained of darting pains through the temples. The stupor and prostration were more marked than on the preceding days.

The abdomen supple, severely painful on pressure.

*Feb. 13th.*—The patient was delirious, and talked a good deal through the night. She was lying on her back; the eyes neither red nor suffused; frontal headach. The central part of the tongue yellowish, deficient in moisture. The abdomen sonorous, distended, painful on pressure. Two liquid stools during the last twenty-four hours. The pulse one hundred and twenty-four, small, felt with difficulty; the heat moderate; the skin dry; dry cough; the respiration laborious; bronchial râle.

*Feb. 14th.*—The patient groaned a good deal; the pulse one hundred and thirty-four; the abdomen distended; one liquid stool.

*Feb. 15th.*—The patient delirious, talked without attending to surrounding objects; the cheeks red; the eyes neither red nor suffused; the pulse one hundred and forty-four, feeble. The patient, when spoken to, put out her tongue, which was moist. She complained when the abdomen was touched. She became more prostrated; the delirium continued.

*Feb. 16th.*—The urine was drawn off by a catheter, and death took place February 17th, at 1 o'clock, A. M.

*Autopsy seventeen hours after death, in cold and dry weather.*

*Exterior.*—Emaciation inconsiderable; stripes on the back; cadaverous rigidity not marked; no eruption.

*Head.*—Pacchioni's glands slightly developed; the veins of the brain distended with black blood; the pia mater moderately injected; the arachnoid transparent; no arachnoid infiltration; no effusion into the ventricles; one or two ounces of clear liquid at the base of the brain. The cortical and medullary substances firm, of a natural colour; the medullary dotted with bloody points. The cerebellum, the corpora striata, the medulla oblongata not remarkable.

*Chest.*—No effusion. The lungs free from adhesions, crepitating, grayish, sufficiently firm. The posterior part of the lower lobes of both lungs of a violet red colour; more friable than other parts of the lungs; not granulated; and, on cutting into them, a considerable quantity of a reddish, frothy liquid, flowed out. The mucous membrane of the bronchi red, neither thickened nor softened. At the summit of the left lung were two or three whitish-yellowish bodies, of the size of the head of a large pin, friable. There was nothing remarkable about the heart, except a spot of an irregular form, and a yellowish colour, just below one of the mitral valves, not to be removed by scraping, on the lining membrane of the heart. One or two similar spots were found in the aorta. The right cavities of the heart contained a considerable quantity of blackish blood, with a fibrinous clot as large as the end of the little finger.

*Abdomen.*—No effusion. The small intestine contracted. The stomach contained a small quantity of liquid, and some mucus. Its mucous membrane was thrown into folds, injected along the great curvature, softened in the great cul de sac, and near the pylorus, so that no strips could be obtained from it. Elsewhere the strips were of proper length. The small intestine contained a moderate quantity of a yellowish liquid. The valvulæ conniventes were marked; the mucous membrane grayish, sufficiently firm. At one foot from the ileo-cœcal valve, opposite the mesentery, was found a patch an inch in length, raised above the surface,—the mucous membrane over it being soft and removeable by scraping with the knife. At an inch from the valve was another patch, from twelve to fourteen lines in length, raised above the level of the surrounding mucous membrane, and in the centre of it an ulceration, with a diameter of three or four lines; the edges raised, the bottom of it on the sub-mucous cellular tissue.

No ulceration on the valve, nor in the cœcum. In the first foot of the colon were six ulcerations, of from four to six lines in diameter. The muscular fibres were not laid bare at the bottom of any of them. The large intestine contained a moderate quantity of yellowish fœcal matter, moderately consistent. The solitary glands were slightly developed. The mucous membrane of the large intestine grayish, sufficiently firm; no ulcerations beyond the first foot. The mesenteric glands were of a rose colour, moderately developed. The spleen was three inches and nine lines in length; its tissue was of its usual firmness. The liver was of its usual size, of a deep red colour, and on pressing it, a considerable quantity of blackish blood flowed from it. The gall-bladder contained from one to two ounces of a viscous, blackish liquid. The iliac veins and the vena cava contained black blood, their lining membrane smooth. In the right iliac fossa, and above the psoas muscle, was a tumour, from which flowed four ounces of pure pus, unmixed with blood. The walls of this abscess were lined with a false membrane, which felt to the finger like a mucous membrane. No communication could be traced between it and any other part. The genito-urinary organs were natural.

Can there be any doubt as to the character of the disease of which the patient died? Can it be any thing else than typhoid fever? In fact, we have the headach, the dizziness, the loss of strength, the stupor, the delirium, the tenderness of the abdomen, the meteorism, the diarrhœa, which are all prominent symptoms of typhoid fever. The lesion which has been considered characteristic of that disease, was present; there were other lesions, but they were evidently secondary, nor did they give rise to any prominent symptoms, or modify appreciably the course of the disease. Let us now take another observation, and compare the two.

SECOND OBSERVATION

Eliza Fightly, a widow, 58 years of age, chesnut hair, hazle eyes, a brown skin. Her muscular system moderately developed; her embonpoint not remarkable; her health habitually good.

Her present illness commenced the 26th of January, by chills, followed by a hot skin, headach, pain in the back and limbs, thirst, inappetence. Some medicine was given to her, and she had three or four liquid stools daily, from the commencement of her illness. She has kept her bed from the first day, and was received at the fever hospital Feb. 7, 1839.

*Feb.* 8.—The countenance pale, its expression not remarkable; the eyes neither injected nor suffused; restlessness; the patient often changed her posture. The headach continued three or four days; she has not suffered from it since. No dizziness, no giddiness, no ringing in the ears; the night quiet, without delirium. The answers to questions exact, but the prolonged exercise of the mind fatiguing and irksome. No pain of the limbs.

The tongue brownish, yellowish, dry, badly protruded; the teeth incrusted; thirst; anorexia. The abdomen well formed, supple, not painful on pressure. No gargouillement; the spleen not felt below the ribs. Three liquid stools in the last twenty-four hours. On the chest and abdomen were numerous spots of a dark red colour, not raised above the surface of the skin, imperfectly disappearing on pressure, of the size of the head of a pin or of a small pea, grouped together. The spots were not so numerous, nor so thickly clustered, on the limbs. The pulse one hundred and ten, slightly developed; the skin hot, dry, no sudamina. The respiration accelerated, infrequent cough.

℞. Mustard poultice to the sternum; four ounces of sherry wine.

*Feb.* 9.—The cheeks red; the eyes neither red nor suffused; drowsiness; prostration more marked; the patient answers with difficulty; no delirium in the night. The tongue brownish, dry; the abdomen supple, well formed, patient did not complain when the strongest pressure was made on it. No gargouillement nor liquid stools. The pulse one hundred and eight; the skin dry; no sudamina.

The patient continued much in the same condition the following days. The abdomen was supple, never painful on pressure; the stools were liquid, two or three in the twenty-four hours.

*Feb.* 10.—The pulse was one hundred and sixteen.

*Feb.* 11.—One hundred and twenty.

The 10th somnolence was noted; complete prostration, &c. 11th, the patient not attending to things and persons about her. Death took place Feb. 12th, at six o'clock, A. M.

*Autopsy eight hours after death, in mild and damp weather.*

*Exterior.*—Cadaverous rigidity well marked; the body not perfectly cold; stripes on the back. Emaciation not marked. The eruption somewhat faded: on cutting through the skin, the red colour extended to the subdermoid cellular tissue.

*Head.*—The dura mater not remarkable, the veins of the brain moderately distended with blood. The pia mater moderately injected. About two ounces of subarachnoid infiltration. The arachnoid over the posterior lobes, and at the base, in spots clouded, not perfectly transparent. From an ounce and a half to two ounces of clear fluid in each lateral ventricle. The cortical substance of the brain of its usual consistence and colour. The medullary substance dotted with blood, firm. The cerebellum, corpora striata, medulla oblongata natural.

*Chest.*—No effusion. *Heart.*—The right cavities contain from one to two ounces of black blood, and in the ventricle was a small, firm, fibrinous clot. Greatest thickness of walls of left ventricle eight or nine lines. Its lining membrane smooth, the valves natural. The aorta not remarkable.

*Lungs.*—Strong adhesions of right lung, which was of a gray colour; crepitating, sufficiently firm; an inconsiderable quantity of liquid flowing out on the section of it. The left lung of a lighter colour, crepitating throughout. The mucous membrane of the larger bronchi injected in bands of a deep red colour, lined by a considerable quantity of mucus, neither thickened nor softened.

*Abdomen.*—No trace of fluid in the abdominal cavity, no meteorism. The stomach contracted, contained only a moderate quantity of mucus. Its mucous membrane natural, except near the cardiac orifice in a square surface of an inch and a half, where it was of a delicate rose colour, mamelonated, and had scarcely more than the consistence of mucus. The small intestine contracted, containing little more than mucus and bile. Its mucous membrane generally grayish, of a rose tint in the ileum, had every where its proper consistence and thickness. Peyer's patches and Brunner's glands, were hardly distinguishable. The mucous membrane of the cœcum, and of the first half of the colon, grayish, and of a consistence scarcely greater than that of mucus. The large intestine contained a moderate quantity of yellowish fœcal matter. The mesenteric and mesocolic glands natural.

The liver natural. The gall bladder contained two or three ounces of a blackish, viscous fluid. In the fibrous coat of the spleen was a cartilaginous spot, an inch square in extent. The size of this viscus was not remarkable. The kidneys and bladder natural.

Was the disease of this patient the same with that of the subject of the first observation?

There certainly are remarkable differences in the histories of the two cases. In the first were certain abdominal symptoms; pain on pressure, meteorism, which were wanting in the last, in which case, also, there was no lesion of Peyer's glands, and this absence of the characteristic lesion of typhoid fever is the striking feature in the case. We may remark, also, the absence of dizziness and ringing in the ears. The affection of the mind, the delirium in the latter part of the disease, the somnolence, are all symptoms of typhoid fever, nor is there anything very peculiar in them as existing in this case.

The age of the subject exceeds the limits given by M. Louis as those of the subjects of his observations.

The eruption certainly was very different from that found in typhoid fevers. Judging from these two cases, should we not be disposed to admit the existence of two forms of continued fever? the second being distinguished from the first by the absence of abdominal symptoms, of the lesion of the glands of Peyer, by the presence of a peculiar eruption, and by a liability to the disease on the part of older persons.

Of the thirteen observations on which this memoir is based, we can have no hesitation in referring four of the fatal cases to the second form, and we may safely place five of the convalescents in the same category.

Under the other form we shall have one fatal case, and two cases where the patient recovered.

Let us pass at once to the analysis of these observations.

Art. I.—*Age, sex, residence of patient, march, duration, termination of the disease, causes.*

The three patients of the first series were all females. One forty-one years of age, the second twenty-two years, and the third twenty years. Two of them had always lived at London, one had been there eleven years. Of the second series there were four females and three males. Two of these patients had always resided in London, one had recently arrived there. Two of these patients were fifty-eight years of age, one forty, one fifty-one, the other three were between twenty and thirty. The average age of the patients of the first series was twenty-seven, that of those of the second series forty-one. This difference is remarkable, and we should remember, also, that the average age of the subjects of M. Louis' observations was twenty-three, and that no patient had reached the age of forty. Nearly all his patients had been in Paris but a short time.

The patients in both our series were healthy. One only had been unwell for some weeks. One patient was addicted to alcoholic excesses. One of the first series, and two of the second, had taken care of persons who were believed to have fevers. The disease commenced in both series at different hours of the day. The first symptoms were chills, not repeated in any case after the first day, followed by heat inappetence, thirst, headach, pain in back and limbs.

Weakness was an early symptom in the patients of both series. The symptoms from the senses came next in order, and delirium and somnolence came on at an advanced period of the disease. There were not distinct periods of the disease in any case. One of the cases of the first series, and four of the second terminated fatally. One of the patients of the second series died on the 10th day, one on the 18th day, one on the 20th, and one on the 29th.

One of the patients of this series was convalescent after an illness of six weeks. A second was regarded as convalescent the 22d day. Of the first series one case terminated fatally the 18th day, and in one case convalescence commenced the 27th day.

Art. II.—*Cerebral Symptoms.*

*Headach.*—In all the cases of both series the headach was one of the first symptoms. In the fatal case of the first series it existed the twelfth day, was lancinating, though less violent than at the commencement; in one of the other cases it existed the eighth day, in the third case it continued three days only. In one case of the second series the headach continued three or four days. In a second case the headach did not exist at the commencement of the fourth week; in a third case the patient complained of a general lancinating headach the seventeenth day, in a fourth the headach continued nine days, and in a fifth it was not present the eighteenth day.

*Sleep.*—The sleep was not natural in any case. In one of the cases of the first series imperfect unrefreshing sleep was one of the first symptoms, and it continued the fourteenth day. Somnolence was observed during two or three days, commencing the sixteenth day. In a second case sleeplessness was noted the eighth day. In the third case somnolence was observed the thirteenth day. In one case of the second series somnolence was observed the sixteenth day. In a second case somnolence was observed the ninth, the patient dying the thirteenth day. In a third case somnolence on the twenty-second day, and continuing during two or three days.

A fourth patient had no refreshing sleep the seventeenth day, was somnolent the eighteenth, was sleepless the twentieth, and slept naturally the twenty-second.

A fifth patient was sleepless for two nights at an advanced period of the disease, then became somnolent, and the somnolence continued for two or three days. In a sixth case the sleep was unrefreshing and disturbed by dreams during the first week, and somnolence was observed for the first time on the eleventh day. In the seventh patient the sleep was imperfect the nineteenth day, somnolence was noted the twentieth, continuing till death.

*Delirium.*—In all the cases the mind was more or less affected, and the exercise of the mental faculties was attended with more or less difficulty. The patient answered correctly but slowly when seen for the first time, but as the delirium came on, the answers were wild, though in many cases answers to simple questions could be obtained by fixing strongly the attention of the patients. In the fatal case of the first series delirium was first noted the sixteenth day. The patient talked a great deal, but was not violent. A second patient was delirious the eleventh day, and quite agitated the fourteenth. In the third case delirium did not exist at any period. In the first case of the second series the delirium was not violent, and supervened three days before death. The second patient was slightly delirious the twelfth day, but answered simple questions the day before that on which the disease terminated fatally. The third patient was anxious about himself, talked incoherently the twenty-second day, left his bed on the night of the twenty-fourth; the delirium then alternated with somnolence, but did not return after the twenty-ninth. In the fourth case there was no delirium, though the patient had a very imperfect use of her mental faculties for some days. In the fifth case a straight jacket was necessary during one day. The sixth patient became delirious the twelfth day, was quite agitated and talked a great deal that night. The eighteenth day he recognised his wife, but the twenty-second he no longer noticed surrounding objects. In the last case the patient was delirious the last day or two of life only, and never violently so.

*Dizziness.*—In the first case of the first series, dizziness was noted the eleventh day, but did not come on with the first symptoms. In the second case this symptom presented itself at the very commencement of the disease, and in the third case it was found on the eighth day. The symptom we are considering was absent in four of the cases of the second series. It was one of the first symptoms in one case.

*Stupor, muscular strength.*—Stupor was noted in the three cases of the first series, and in all but one of the cases of the second series.

*Loss of strength.*—In the fatal case of the first series the patient was obliged to keep his bed after the third day, and the twelfth day the prostration was quite marked. In the second case the patient was not entirely confined to her bed during the first week, the prostration was sufficiently marked at the close of the second week. The third patient kept her bed from the third day, and the prostration was quite marked the eleventh day. In the first case of the second series the patient kept his bed from the first day and the prostration was very marked from the fifteenth. In the second case the prostration was quite marked the tenth day. The third patient was found quite prostrated at the commencement of the fourth week. Two days later, he got up and placed himself on the chair at the side of his bed. The fourth patient kept his bed from the first day. The fifth patient was quite prostrated at the advanced period of the disease when he came under observation. The sixth patient kept about for the first week, was confined to his bed from the tenth day, and the prostration was very marked the eighteenth day. In the seventh case the patient was confined to his bed from the fourth day, was prostrated the tenth day, but got up for his stools.

*Symptoms from the senses.*

1. *Sight and condition of the eyes.*—The eyes were not red nor suffused in any of the cases of the first series. In the two first cases, the sight was confused the first days; in the third case, increased sensibility to light was noted the eighth day. Redness and suffusion of the eyes were noted in two cases of the second series, and in two other cases the sight was indistinct.

2. *Hearing.*—In one of the three patients of the first series, deafness and ringing in the ears were noted. In one case of the second series the hearing was hard the seventeenth day.

*Epistaxis.*—This was found in one of the cases of the first, and in one of the second series.

Art. III. *Symptoms from abdominal organs.*

*Diarrhœa.*—This symptom existed in the three cases of the first series, but appeared as early as the first day of the disease, in one case only. The diarrhœa ceased only with the termination of the disease. No one of the patients had more than three or four liquid stools daily. In one of these cases the stools were involuntary the fourteenth day of the disease. We must, however, take into account the treatment. Thus one patient, we observed, for the first time, at the commencement of the fourth week, had three liquid stools in the twenty-four hours, but was taking a pint of beef tea, four ounces of sherry wine, and pills of the carbonate of ammonia and of camphor. And though diarrhœa existed in every case, it was an early symptom in one case only, and five of the patients had taken laxatives or stimulants. The stools were involuntary in two cases, the last, and the last two days of life.

*Tenderness of the abdomen on pressure.*—The three patients of the first series complained when pressure was made on the abdomen. In one case there was colic at the commencement, and pain, independent of pressure. In six of the cases of the second series, there was no tenderness of the abdomen on pressure; one of these patients complained of a sensation of fulness. In the remaining case there was a general sensibility of the skin, but the patient complained more when pressure was made on the abdomen, than on any other part.

*Meteorism.*—This symptom was found in the three cases of the first series, existing at the time the patients came under observation, and continuing several days.

Meteorism was observed in one of the patients of the second series, and but for one day.

*Gastric symptoms.*—The absence of nausea, vomiting and epigastric pain in all the cases of both series, is worthy of remark. In some of the fatal cases there were lesions of the gastric mucous membrane.

Shall we attribute the absence of symptoms to the delirium?

### Art. IV.—*Tongue and teeth.*

In the first case of the first series the tongue was moist with a whitish coat the eleventh day, and was in the same condition the day before that on which the patient died. In the second case the tongue was red, dry, badly protruded, the teeth encrusted the sixteenth day, having been moist and covered with a whitish coat the preceding days. On the eighteenth day the tongue was moist. In the third case the tongue was dry and covered with a yellowish coat the eleventh day.

In the first case of the second series, the tongue was brownish and dry the fourteenth day, the teeth encrusted, and continued so till death ensued, four days later.

In the second case the tongue was badly protruded, its central part dry and brown the tenth day. Two days later, it became black, and continued so till the disease terminated in death on the fourteenth day. In the third case the tongue was black and dry, at the commencement of the fourth week. In the fourth case, on the seventeenth day, when the patient came under observation, the tongue was dry, black and cracked, the teeth encrusted, and they were in that condition two days.

In the fifth case, when the patient was first observed, the tongue was dry, badly protruded, the lips and teeth were covered with sordes.

In the sixth case the tongue became black and dry, two days before death, and in the seventh case they were found in the same condition the eighteenth day of the disease, the patient dying on the twenty-ninth day.

*Symptoms from urinary organs.*—In one of the cases of the first series, retention of urine was noted on the day preceding that of the fatal termination. In another case the urine was passed in bed without the knowledge or attention of the patient. In three of the cases of the second series, the discharge of the urine was not controlled by the will.

### Art. V.—*Respiration, râles, cough.*

The respiration was accelerated in all the cases of the first series; in two of them there was a cough and a sonorous râle. The respiration was accelerated in all the cases of the second series, and in three of the fatal cases it was more or less embarrassed during the two or three last days of life. Cough and râle were noted in three cases, but in several cases auscultation could not be properly practised, on account of the great weakness of the patients.

### Art. VI.—*Pulse.*

The acceleration of the pulse was nearly the same in the two series of cases. Thus in the fatal case of the first series, on the eleventh day, the pulse was 104, moderately developed; on the fourteenth day 124 and small; on the sixteenth day 144. In one of the other cases of the first series, the pulse was 100, moderately developed on the fifteenth day; 120 on the sixteenth day; and 72 on the twenty-first. In the first case of the second series the pulse was 120 on the fourteenth day, and on the sixteenth it was small and feeble. In another fatal case, the pulse was 120 and small on the nineteenth day, and on the twenty-ninth it was 144. In one case which terminated favourably, the pulse was 90 at the end of the third week; three days later it had risen to 120, and three days later it fell to 90. The pulse was regular in all the cases.

### Art. VII.—*Skin.*

In five of the cases of the second series, we found a peculiar eruption on the limbs as well as on the chest and abdomen. The eruption consisted of spots of rather a dark red colour, small, from the size of a pin's head to that of a pea, irregularly grouped together, disappearing on pressure, but imperfectly after the first day of its appearance, not raised above the surface of the skin. The precise date of the appearance of the eruption could not be determined in all the cases, for it was found on the entrance of the patients into the hospital. In the fatal cases it continued till death, and in two cases, where the patients recovered, in one it was found on the twenty-fifth day, and in the other on the thirty-first day.

In two of the cases of the first series, there was no eruption; in the third case the rose spots and the exanthematic eruption both were present. The observation is imperfect, but as it is interesting in this connection, I submit it to the reader.

(*To be continued.*)

